# Just visiting: A qualitative study of Australian allied health professionals' experiences working in residential aged care facilities during the COVID‐19 pandemic

**DOI:** 10.1111/ajag.13217

**Published:** 2023-06-15

**Authors:** Isabelle Meulenbroeks, Karla Seaman, Magdalena Z. Raban, Nasir Wabe, Johanna Westbrook

**Affiliations:** ^1^ Australian Institute of Health Innovation Macquarie University Sydney New South Wales Australia

**Keywords:** allied health occupations, COVID‐19, qualitative research, residential aged care

## Abstract

**Objectives:**

Internationally, the COVID‐19 pandemic has negatively impacted health professionals' experiences of delivering effective care. The experiences of health professionals are important: poor experience has been associated with poorer patient outcomes and high staff turnover. This study aimed to narratively explore the impact of the COVID‐19 pandemic on the experience of delivering allied health (AH) care in Australian residential aged care (RAC).

**Methods:**

Semistructured interviews were conducted in February–May 2022 with AH professionals who had experience working in RAC during the pandemic. Interviews were audio‐recorded, transcribed verbatim and thematically analysed in NVivo 20. Twenty‐five per cent of interview transcripts were independently analysed by three researchers to create a coding structure.

**Results:**

Three themes were identified from interviews with 15 AH professionals to describe experiences delivering care pre‐COVID‐19, during COVID‐19, and perceptions of care delivery in future. Prepandemic AH in RAC was believed to be under‐resourced, delivering low‐quality and reactive care. During the pandemic, pauses in, and the slow resumption of, AH services exacerbated professionals' feelings of being undervalued in resident care and in the workforce. Participants were optimistic about the impact AH could have in RAC in future if practice was embedded, multidisciplinary and funded appropriately.

**Conclusions:**

AH professionals' experiences of delivering care in RAC are often poor, regardless of the pandemic. Further research on multidisciplinary practice and health professional experience in RAC is needed.

**Practice Impact**

This study highlights the poor experiences AH professionals have working in RAC, which has only been exacerbated by the pandemic, and the urgent need for AH‐inclusive research and policy in RAC in future.

## 1 INTRODUCTION

Allied health (AH) services, such as occupational therapy, dietetics and physiotherapy, are critical to maintaining the function and well‐being of older adults in residential aged care (RAC) facilities. Internationally, it has been demonstrated that higher ratios of physiotherapy and occupational therapy staffing have been associated with a reduction in falls and higher facility quality ratings^1^, ^2^, ^3^ and access to dietitians has been associated with being well‐nourished.^4^ Currently, in Australia, industry reports suggest that levels of AH service delivery and accessibility are insufficient to meet resident needs.^5^, ^6^ Notable forces driving low levels of AH delivery in RAC include limited funding for AH, unequal distribution of the AH workforce (e.g. between rural and metropolitan areas) and difficulty in attracting and retaining workers.^1^, ^7^, ^8^

Since 2020, health systems globally have been challenged by the coronavirus disease 2019 (COVID‐19) pandemic. However, the impact of the pandemic in RAC was amplified. First, RAC facilities provide care to people who are among the most vulnerable to negative outcomes of the virus.^9^ Second, Australian RAC services were ill‐equipped to cope with additional pressures, from the virus or containment measures (i.e. visitor restrictions/facility lockdowns, isolation within the facility and of unwell staff, personal protective equipment [PPE]) due to long‐standing issues such as low staffing ratios, inconsistent governance and low funding.^10^, ^11^ As a result in Australian RAC, COVID‐19 caused critical nursing and care staff shortages, reduced care activities in facilities and directly and indirectly contributed to poorer resident outcomes.^12^ Cumulatively, this has taken a toll on the people providing care with RAC workers reportedly suffering high rates of burnout, stress and fatigue.^13^

There is a growing body of evidence that has associated negative health professional experiences with increased errors, poor patient safety and workforce exit.^14^ Internationally, the additional pressures of COVID‐19 have increased health‐care worker exit with studies in the United States reporting that up to 40% of health professionals intend to leave the industry in the next 2 years.^15^, ^16^ This is particularly concerning for the RAC sector, as Australian facilities were already experiencing a 29% annual turnover of staff prepandemic.^7^ The importance of the health professionals' experiences is summarised in local Australian State strategic goals, the quadruple aims, where it is believed satisfied employees underpin improved patient outcomes, patient experience and cost‐effectiveness.^17^

COVID‐19 is likely to have had a unique impact on AH professionals in RAC. Industry reports suggest that there were significant pauses in care provision and a reduction in RAC AH service delivery. AH service delivery disruptions may have been caused by COVID‐19 restrictions, such as restricted RAC access (some facilities denied access to all external visitors), telehealth adoption, isolation requirements and redirection of AH staff away from AH roles during staffing pressures.^18^, ^19^ During this time period, service delivery may have also been disrupted as a result of an announcement of a new funding structure for Australian RAC. The new funding instrument does not consider AH services in its structure.^20^ The experiences of AH professionals working in this environment have not yet been explored. This study adopted an explorative qualitative design to gain a narrative understanding of AH professionals' experiences working in RAC, the impact of the pandemic on these experiences and the perceptions on the future of their practice postpandemic.

## 2 METHODS

### 2.1 Design

**FIGURE 1:**
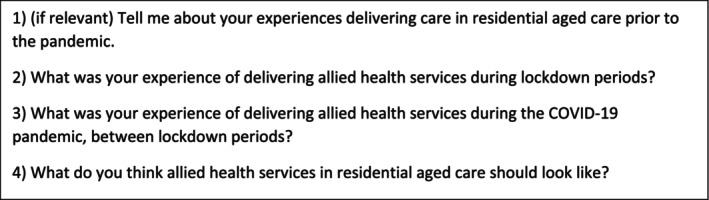
Interview schedule questions.

The consolidated criteria for reporting qualitative research (COREQ) was used to design and report this qualitative study.^21^ The study used semistructured one‐off interviews to explore AH professionals' experiences delivery care in RAC to allow flexibility for participants to share their varied experiences. The interview guide (Figure 1) was developed by the research team and was piloted with a convenience sample of AH professionals prior to use. The interview guide was designed to explore experiences pre‐ and during COVID‐19 and perceptions of the future of AH in RAC. It followed the questions outlined in Figure 1 with additional prompts to explore concepts as needed. The project received ethics approval from the Macquarie University Medicine and Health Sciences Human Research Ethics Subcommittee (ID: 10995).

### 2.2 Recruitment and data collection

Participants were conveniently sampled. They were recruited through leading AH organisations, RAC workforce organisations, RAC providers, AH employer groups and social media (Facebook, Twitter and LinkedIn). Recruitment continued from February to May 2022.

At this time, many Australian jurisdictions had begun to relax COVID‐19 restrictions in the community, which had been in effect in varying levels since the onset of the pandemic (e.g. travel between states, mask wearing, social distancing and testing requirements). However, restrictions such as limited number of visitors, COVID‐19 testing, isolation periods and mask wearing continued in RAC. Also, nationally, the sector had, in February 2022, just passed the peak of the largest COVID‐19 wave in RAC to date.

Eligible interview participants were AH workers who had experience in the RAC sector, for example clinical, managerial and policy experience, during the pandemic (defined as being from March 2020 to current). Participants could have experience in RAC prior to this, but it was not necessary for participation. Internationally, there is no clear definition of AH. Some Australian government and professional organisations define allied health using a specific list of professions, whereas others use the term as an umbrella term for professions who fall outside medical and nursing fields.^22^, ^23^ In this study, eligible participants needed to self‐identify as an AH professional. Nurses and doctors were excluded.

Interviews were conducted over phone or Zoom at the participant's choosing. All interviews were conducted by one researcher (first author) who has experience conducting interviews in similar groups^24^ and is an AH professional. The researcher had no pre‐existing relationship with any of the participants, and no incentive was provided to participate. All participants chose to participate either because they were interested in the topic, were motivated to participate by colleagues or wanted to support research endeavours. A reflexive journal was kept by the interviewing researcher postinterview to document key concepts.

All interviews were audio‐recorded and transcribed verbatim using Otter, a web‐based artificial intelligence transcribing service.^25^ The interview transcripts were manually checked by a researcher to de‐identify participants and check transcription accuracy. Recruitment continued until thematic saturation was reached, when no new themes were raised in four consecutive interviews after reaching 10 interviews.^26^

### 2.3 Analysis

Interview transcripts were coded using thematic analysis^27^ in NVivo 20.^28^ Thematic analysis followed six steps: content familiarisation, initial coding, generation of themes, review of themes, definition of themes and write‐up.^27^ Three researchers independently familiarised themselves with transcripts and conducted initial coding on 25% of the transcripts. A coding structure and themes were created in subsequent team meetings. All researchers involved in the analysis had clinical and research experience in residential aged care settings. One researcher continued to use and develop the coding structure for the remaining transcripts. Changes to the coding structure and theme development were discussed in regular team meetings. Participants did not check transcripts or provide feedback on results.

## 3 RESULTS

Fifteen AH workers participated in an interview. The median length of interviews was 25.56 min (range 18.06–57.02). All participants were women, an average of 38 years old, and most worked in New South Wales or Victoria (*n* = 10, 67%; Table 1).

**TABLE 1:** Participant demographics.

Female (count)	15
Age (years) (median, range)	38 (25–66)
Experience in AH (years) (median, range)	10 (1–40)
Experience in RAC (years) (median, range)	7 (1–30)
State (count)	
NSW	5
VIC	5
QLD	2
SA	1
WA	1
Professional group (count)	
Speech pathologist	6
Physiotherapist	3
Diversional therapist	3
Psychologist	1
Occupational therapist	1
Dental practitioner	1

Three themes were identified in the thematic analysis (1) AH in RAC: at baseline already stretched thin, (2) The COVID‐19 pandemic: An unremitting pressure cooker event, and (3) Into the future. The coding structure, including major and minor themes and quote examples, is presented in Appendix S1.

### 3.1 Allied health in RAC: At baseline already stretched thin

In the first theme, participants described RAC as a system plagued by systemic issues which are driven by perpetual under‐resourcing. In relation to AH, underfunding was thought to be a driver of largely assessment‐only AH care rather than care interventions. Furthermore, participants often described the few interventions that were applied as reactive, rather than preventative, and non‐evidence‐based, like massage for pain relief. Underfunding was also believed to have exacerbated AH workforce issues including the highly contracted, part‐time and siloed workforce structure which AH normally operates in. This limits continuity of care for residents and multidisciplinary practice in facilities:

I used to see people I would think… If we can just practice this transfer for five days in a row, you'd probably be able to do it independently, and then you wouldn't need someone in the room …. And then I look at what I have to do the next day, and I wouldn't be able to fit them in until six days' time.(Participant 1, Physiotherapist)

Participants also believed that the under‐resourcing of other multidisciplinary team members impacted residents' ability to achieve AH goals, such as regular mobility, as care staff do not have the resources, skills, or supportive environment to facilitate regular activity. Overall, participants felt that their role in RAC was not seen as important by other RAC staff, managers and policymakers. Their role was not seen as important and was the first place to cut budgets and staff when finances became strained.

Due to their limited role and recognition in RAC, participants described their work experience as ‘soul destroying’, ‘disheartening’, ‘dispensable’ or ‘disposable’. Participants also believed that AH work in RAC was sometimes non‐engaging, lacked a career pathway or commitment to workforce development and was poorly remunerated compared to community and acute care. Most participants described themselves and their colleagues as working out of a sense of duty to care for older adults. Consequently, it is difficult to attract people to the workforce:

The people who are in aged care are in it for the love of it. It's not for the money. [It's] because of their sense of purpose but also their sense of meaning. [It's] because if they don't do it then no one else will.(Participant 14, Speech pathologist)

### 3.2 The COVID‐19 pandemic: An unremitting ‘pressure cooker’ event

Participants described the pandemic as a ‘pressure cooker’ event that placed additional stress on an already stretched system. For AH workers, stress initially arose from facility lockdowns. AH workers were seen as visitors and restricted from entering facilities and volunteers—who many participants relied on to assist in delivering AH care, for example to help residents attend group sessions—were no longer able to visit. As a result, AH services ceased or significantly reduced. Stress also arose from PPE and testing resource constraints where some AH workers described spending extensive time sourcing their own PPE and COVID‐19 tests. Many workers also believed that full PPE made it difficult to communicate with residents, particularly those with hearing difficulties, and impacted resident‐clinician rapport:

I have to do a RAT [rapid antigen test] test at each facility. Couple of things to note there. One day I visited four residential aged care facilities [including one resident] who might die and had to do four RAT tests.(Participant 13, Psychologist)

The impact of the pandemic was not contained to periods of outbreak or facility/community lockdown. It was felt that restrictions were imposed in RAC beyond a logical period which kept AH service delivery to a minimum for longer than required from an infection control perspective. Ongoing nursing shortages in the sector also impacted AH services:

There was only that time, that six week time, where the facilities would allow anyone in. But then after that time when the restrictions eased by the state government, it was still a hard slog for me to get people and volunteers and entertainers back in all the rest of it.(Participant 7, Occupational therapist)

Some participants described that, once the strictest of restrictions eased, they received an influx of referrals and experienced long wait lists as they sought to provide care for those who had missed routine care during the pandemic and those who had an increased need for AH services during this period.

To adapt to fluctuations in demand and COVID‐19 restrictions, many AH professions reported modifying their practice. A major modification was the transition to telehealth. Other modifications to care delivery included prioritisation of workload, delivery of independent activity packs, use of electronic health records to remotely review residents, increased one‐to‐one sessions, smaller group sessions and socially distanced therapy:

But in terms of all of those referrals that came through, the things that were deemed less urgent, actually on a risk scale, not because they're unimportant… they were missing out on all of that input in that critical time.(Participant 12, Speech pathologist)

The impacts on practice, across all areas of aged care inclusive of AH, were believed to have negatively affected residents. Poorer mental health and increased behavioural problems due to facility lockdowns and isolation in general were frequently mentioned by participants. Other outcomes such as malnutrition, pneumonia, poorer mobility, oral hygiene, quality of life, pain control and a higher mortality rate were specifically attributed to disrupted AH services.

For participants, the increased pressures of the pandemic were often faced without perceived support (e.g. mental health services for staff, additional tools to support care, additional sick leave to account for time spent off work for COVID‐19 symptoms, or financial support for AH workers who lost their income). Participants found that teamwork, supportive management and focussing on positive achievements (e.g. technology acceptance and building a stronger connection with residents) helped during this time. But many participants overwhelmingly felt the experience of working in RAC during the pandemic was negative. The experience confirmed their perceptions that AH workers are dispensable and not a part of the RAC workforce:

I think COVID has detrimentally influenced how allied health is delivered in aged care and the recognition that we receive in aged care, and that's reflected by the delays in service provision, during the pandemic and then how the facilities have responded to that following the recommencement of services.(Participant 10, Speech pathologist)

### 3.3 Into the future: multidisciplinary, preventative care

Aside from the pandemic, in recent years pressure has also been arising from other sources such as the change of funding structure in October 2022—causing uncertainty about the funding for AH in future: low remuneration and higher incentives to work in other areas such as the National Disability Insurance Scheme (NDIS). Participants reported that these pressures were causing workforce shortages and led some to exit their own role as an AH professional in RAC:

Number one, we're burnt out that's why I left you know, we're burnt out and we don't have the support and there's not the resources, unfortunately.(Participant 14, Dietician)

Despite often expressing dejection about current AH services in RAC, participants were hopeful about the potential for AH in RAC. Participants believed that in order to achieve its potential, AH workers should be embedded in every facility rather than contracted. This in‐house model of care was believed to foster multidisciplinary practice and continuity of care, improving resident care quality and workforce issues. However, some pointed out that this may not be feasible for all facilities due to size and rurality.

Participants believed that in order to achieve any of the improvements they suggested to AH service delivery in RAC, AH required more funding. The funding would also be required to support workforce retention strategies and development, access to and affordability of AH to consumers and improve facility environments and resources to promote safe activity and participation:

But we're the people who put the quality into the lives of people. We provide that to them to enrich, essentially help, people achieve goals. I think there's a really deep need for it, but whether or not that, you know, they're not really, really willing to spend the cash.(Participant 3, Diversional therapist)

In future lockdowns, participants wanted to see AH services proactively deployed to prevent deterioration in resident outcomes due to isolation. Many of the modifications to practice adopted during the pandemic (e.g. telehealth) were considered inappropriate for this population, no substitute for face‐to‐face care, and use should be limited in future. In future lockdowns, participants also wanted to see greater inclusion of AH in the workforce and recognition of residents' well‐being and rights with fewer strict lockdowns. Participants also believed that RAC could be better‐prepared in future with PPE, tests, consistent visitor regulations and improved environmental layout. While participants had strong opinions about how AH services in RAC could be delivered to improve resident outcomes and support the broader RAC workforce, many believed that realistically the current status quo would never change.

## 4 DISCUSSION

The experiences of AH service delivery in RAC during the pandemic left many participants with an overarching view that AH is not considered essential in RAC by the multidisciplinary team and policymakers. While the pandemic provided tangible examples of this (e.g. being viewed as a visitor or having limited support), it simply exacerbated the pre‐existing situation which was largely driven by low funding and the broader RAC environment. Experiences shared during interviews suggest that during COVID‐19, AH care may have been delayed or missed and that these impacts may have a lasting effect on the AH workforce.

Australian RAC facilities enacted comprehensive COVID‐19 procedures to protect older adults during the pandemic. While these were necessary, participants believed the restrictions extended for too long and often failed to consider AH service delivery and the AH workforce. Participants had few positive experiences that may be re‐deployed in future crises or continued postpandemic. Changes to practice such as telehealth or individual activities were thought not to be appropriate for the RAC population. This is consistent with the finding of a survey of 868 Australian AH professionals (in multiple settings) where 40% believed the quality of care was poorer with telehealth consultation.^19^ Instead, to improve performance in future times of crisis, participants believed that underlying issues in RAC needed to be addressed.

Funding was a key issue raised by participants; AH practice was limited by funding prepandemic. AH received limited funding support in the COVID‐19 response, and the funding structure was going to change in October 2022. The outgoing funding instrument, the Aged Care Funding Instrument (ACFI), provided minimal funding for AH mainly in the form of massage for pain relief, was replaced by the Australian National Aged Care Classification funding model (AN‐ACC) which provides no direct funding for AH. A survey of 279 AH professionals in late 2022 demonstrated that the funding restructure has caused 13% of participants to lose their job, 43% to lose hours and 30% to consider leaving their role in RAC.^20^ AH certainly requires more than no funding, but it is uncertain how much AH should receive or how funding and AH service provision should be monitored. A suggestion to the Royal Commission of Aged Care Quality and Safety was the inclusion of AH in target care minutes per resident per day.^29^ Such benchmarks are already in place internationally. For example, Ontario in Canada set a target of 36 min of AH service per resident.^30^ However, there is limited evidence of the impact this has on clinician experience or resident outcomes. More clarification regarding AH funding is required. Without it, as evidenced in these interviews and industry reports, AH professionals continue to feel undervalued.

Participants in this study believed that AH was frequently actively excluded from the RAC workforce. For participants, this was demonstrated in facility lockdowns where some AH professionals could not visit clients, the slow recommencement of services, and government workforce strategies such as a 15% pay increase for RAC staff and a once‐off ‘thank you’ pandemic payment. This experience is also likely unique to the RAC setting, as AH services in other settings were frequently embedded in emergency planning and COVID‐19 protocols. For example, in hospitals, AH professionals were considered vital in the management of people with COVID‐19, supporting rehabilitation efforts and early discharge, and were involved in surge workforce plans such as through upskilling or skill sharing with other professional groups.^31^, ^32^ The exclusion of AH in RAC may be a result of the limited multidisciplinary practice in this setting. While multidisciplinary teams are the gold standard in acute care settings, their application in RAC is limited. There is also limited evidence to support their uptake^33^ beyond findings that multifactorial interventions are often best practice in RAC; for example, exercise, equipment prescription and staff education (which AH can support) in combination can prevent falls.^34^ The evidence gap, and limited practice of multidisciplinary care, creates misunderstanding of the value AH professions could have in RAC within the team, facility and policy environment. Excluding professions in the process to reform RAC is not constructive. As research and practice evolves in RAC, professional groups must recognise that the advancement of all persons in RAC is mutually beneficial for all team members and for resident care.

This study is unique as it is the first Australian study, to our knowledge, to qualitatively explore the experiences of AH professionals in RAC. However, it has limitations. AH in RAC is a large and diverse group. Not all professions are appropriately represented in this study (e.g. AH assistants who make up approximately 50% of the AH workforce in RAC).^7^ Regardless, while individual disciplines had nuances specific to their discipline, thematic saturation was reached during the recruitment process. All participants were female. However, this is closely reflective of the industry; approximately 88% of the AH RAC workforce are female.^8^ Participants in this study are also likely to feel more strongly about the subject, than those who did not participate. Therefore, these interviews may over‐represent discontent in working conditions in RAC and experiences during the pandemic. Lastly, due to recruitment methods it is unclear how many people were aware of the study but chose not to participate.

## 5 CONCLUSIONS

The experience of working in RAC during the pandemic left participants feeling that their AH role was seen as non‐essential to resident care and in the workforce. Many drivers of negative AH professional experiences, including low funding and exclusion from the workforce, existed prepandemic. The pandemic and the recent funding change likely only exacerbated pre‐existing factors driving negative experiences and provided tangible examples. To improve AH professionals' experiences, and with it resident care, more data and research are urgently needed in RAC multidisciplinary care and workforce experience. More broadly, the sector also needs to include all health professions in reforms as there is no progress if team members are left behind.

## CONFLICT OF INTEREST STATEMENT

No conflicts of interest declared.

## Supporting information


Appendix S1

## Data Availability

The data that supports the findings of this study are available in the supplementary material of this article.
