# Supplementary material for: Just visiting: A qualitative study of Australian allied health professionals' experiences working in residential aged care facilities during the COVID‐19 pandemic
Source: Australas J Ageing. 2023 Jun 15;42(4):690–7. doi: 10.1111/ajag.13217 (PMC10946948; doi:10.1111/ajag.13217)
Supplement: Supplementary file 1 — Appendix S1 [file AJAG-42-690-s001.docx]

# Appendix 1

**Table 1. Theme 1: A stressed system coding structure**

| **Subtheme name** | **Description** |
| --- | --- |
| Aged care staff general | Participants highlighted how aged care staff could help facility therapy activities day to day between therapy however this practice is currently limited due to insufficient education/training, lack of expectation or time, and understaffing. Participants did also recognise that this workforce is overworked, underpaid, and, like them, doing the best they can with limited resources. |
| Allied health staff | Currently in residential aged care, allied health professionals find the work limiting, non-engaging, and experience a lack of recognition. Participants acknowledge that even at baseline there is high intent to leave the industry. However, they are there because they strongly believe that residents deserve better. They are there for the love of it, sometimes dedicating their own time to source funding and performing advocacy roles. |
| Residential aged care; a unique hospital | Participants described the current model of care as reactive, task-orientated (particularly in nursing disciplines), and siloed. While some participants described care in aged care as hospitalised, some participants also drew parallels from acute or subacute care where the accepted norm of allied health service delivery is higher. |
| Resident quality of life and outcomes | In the stressed system residents have less than ideal outcomes including deteriorating independence and poor quality of life. In this code participants often discuss real and/or hypothetical cases of current care where residents experienced poor outcomes. |
| Systemic issues | Participants describe that the current care delivery model is insufficient; it is underfunded, access is complex, encourages inconsistent staffing, services are limited and only available for the highest priority. For some professions there is also a lack of recognition and funding in the broader community (i.e., dentistry) causing many carers, families, and residents to be uneducated regarding the need for the service. |

**Table 2. Theme 2: COVID-19, a ‘pressure cooker’ event coding structure**

| **Subtheme name** | **Description** |
| --- | --- |
| Immediate consequences of the pressure cooker | Several participants described that once pressure increased in the sector, allied health was considered an optional extra. Also, as a result from the increased pressure, some residents missed allied health services, and this may have disproportionally impacted some groups. A major impact on service delivery during COVID-19 for some allied health professions was the introduction/change to telehealth which was often described as being inappropriate for this population. Prioritisation was also tightened to manage increased pressure, meaning that only the most significant needs were addressed at this time. |
| Increased pressure | Many participants described a period of increased pressure in their facility. This was caused by a confluence of factors including restrictions which directly limited or banned their practice, nursing staff shortages and increased need. |
| How to survive a pandemic | Some participants mentioned strategies which helped them to navigate the worst of the pandemic and the aftermath. Strategies included supportive management, prioritisation, and increased service delivery. |
| Over the side | As health professionals emerged from the depths of the pandemic in their local context, immediate and more long-term impacts were apparent to some professionals including deteriorated oral and mental health. Immediate service impacts included long waiting lists and increased referrals. There was some impact on intent to leave the workforce along with other general pressures felt in the workplace. |
| The long tail | Unfortunately, past the peak of COVID-19 infections and/or restrictions often did not see a rapid return to normal practice, sometimes even though there were no COVID-19 cases apparent in the facility or the community. Causes of the ongoing impact of COVID-19 on practice included fear, by the staff and residents, and ongoing restrictions on distancing, masks and group activities. |

**Table 3. Theme three: Into the future coding structure**

| **Subtheme** | **Description** |
| --- | --- |
| Pressure arising from other sources | COVID-19 is just one stressor: other things like regular audits and the changing funding structure also generate increasing pressure on the system even at baseline. |
| Access | Participants reported that they felt that preventative or intensive care was neglected once a person became an aged care resident. They advocated that resident should be treated as other citizens elsewhere in the health system. |
| Environment | The layout and set up of residential aged care could be improved by making it more homely and appropriate to the older co-morbid population. |
| Future lockdowns | Participants described what they thought would work in future lockdowns. |
| Meaningful care | Here participants have described how future care needs to be more meaningful i.e., remove group classes, encourage individualised care, maintenance as an appropriate etc. Meaningful care needs to focus on quality of life. |
| Meaningful measures | Participants suggested removing or updating some of the tools used in aged care presently as they have little meaning for staff and residents. |
| Multidisciplinary | 'Therapy' needs to be facilitated by all - including the nursing staff and allied health professionals across various disciplines. All allied health professionals, GPs and nursing staff should work closer together to achieve better resident care. |
| Service delivery model | Participants preferred, in the future, to have a model of care that encouraged consistent staffing such as the in-house model of care. Participants also suggested in day-to-day practice that telehealth should be limited. |
